# Supplementary material for: Instability of Non-Standard Microsatellites in Relation to Prognosis in Metastatic Colorectal Cancer Patients
Source: Int J Mol Sci. 2020 May 16;21(10):3532. doi: 10.3390/ijms21103532 (PMC7279028; doi:10.3390/ijms21103532)
Supplement: Supplementary file 1 [file ijms-21-03532-s001.pdf]

Supplementary Table S1: Elevated microsatellite alterations at selected tetranucleotide repeats (EMAST) and PCR settings.

		Annealing temperature	Product size (bp)	Reagents	PCR conditions
**MYCL1**	Fwd: TGG CGA GAC TCC ATC AAA G-VIC Rev: CCT TTT AAG CTG CAA CAA TTT C	56.0 °C	181	Kapa2G Robust Hotstart PCR Kit	95°C for 3 minutes; (95°C for 15 seconds, 58/60°C for 15 sec, 72°C for 30 sec) X40 cycles; 72°C for 1 hour
**D20S82**	Fwd: GCC TTG ATC ACA CCA CTA CA -NED Rev: GTG GTC ACT AAA GTT TCT GCT	60.0 °C	249	Kapa2G Robust Hotstart PCR Kit	95°C for 3 minutes; (95°C for 15 seconds, 58/60°C for 15 sec, 72°C for 30 sec) X40 cycles; 72°C for 1 hour
**D20S85**	Fwd: GAG TAT CCA GAG AGC TAT TA-FAM Rev: ATT ACA GTG TGA GAC CCT G	56.0 °C	146	Takara ExTaq	(95°C for 3minutes, 56°C for 1 minute, 72°C for 15 sec) X35 cycles; 72°C for 1 hour
**D8S321**	Fwd: GAT GAA AGA ATG ATA GAT TAC AG -VIC Rev: ATC TTC TCA TGC CAT ATC TGC	58.0 °C	237	Kapa2G Robust Hotstart PCR Kit	95°C for 3 minutes; (95°C for 15 seconds, 58/60°C for 15 sec, 72°C for 30 sec) X40 cycles; 72°C for 1 hour
**D9S242**	Fwd: GTG AGA GTT CCT TCT GGC -NED Rev: ACT CCA GTA CAA GAC TCT G	60.0 °C	178	Kapa2G Robust Hotstart PCR Kit	95°C for 3 minutes; (95°C for 15 seconds, 58/60°C for 15 sec, 72°C for 30 sec) X40 cycles; 72°C for 1 hour
